# 5-Bromo-2,4,6-trimethyl-3-phenyl­sulfinyl-1-benzofuran

**DOI:** 10.1107/S160053680802669X

**Published:** 2008-08-23

**Authors:** Hong Dae Choi, Pil Ja Seo, Byeng Wha Son, Uk Lee

**Affiliations:** a Department of Chemistry, Dongeui University, San 24 Kaya-dong, Busanjin-gu, Busan 614-714, Republic of Korea; b Department of Chemistry, Pukyong National University, 599-1 Daeyeon 3-dong, Nam-gu, Busan 608-737, Republic of Korea

## Abstract

The title compound, C_17_H_15_BrO_2_S, which was synthesized by the oxidation of 5-bromo-2,4,6-trimethyl-3-phenyl­sulfanyl-1-benzofuran with 3-chloro­peroxy­benzoic acid, features a trigonally-coordinated S atom. The phenyl ring is approximately perpendicular to the plane of the benzofuran fragment [dihedral angle 75.11 (7)°]. The crystal structure is stabilized by non-classical C—H⋯O and Br⋯Br inter­actions [3.7169 (6) Å].

## Related literature

For the crystal structures of similar 2-methyl-3-phenyl­sulfinyl-1-benzofuran derivatives, see: Seo *et al.* (2007 ▶); Choi *et al.* (2008 ▶).
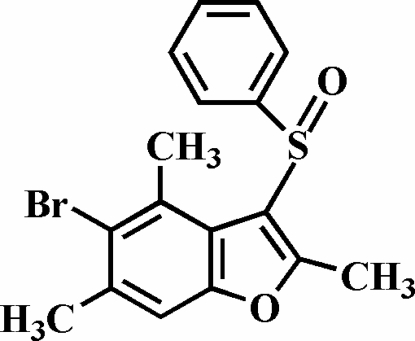


## Experimental

### 

#### Crystal data

C_17_H_15_BrO_2_S
*M* *_r_* = 363.26
Monoclinic, 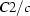

*a* = 22.114 (2) Å
*b* = 10.4281 (8) Å
*c* = 16.675 (1) Å
β = 125.767 (1)°
*V* = 3120.1 (4) Å^3^
*Z* = 8
Mo *K*α radiation
μ = 2.77 mm^−1^
*T* = 298 (2) K
0.30 × 0.20 × 0.10 mm

#### Data collection

Bruker SMART CCD diffractometer
Absorption correction: multi-scan (*SADABS*; Sheldrick, 1999 ▶) *T*_min_ = 0.528, *T*_max_ = 0.762
8646 measured reflections
3055 independent reflections
2607 reflections with *I* > 2σ(*I*)
*R*_int_ = 0.015

#### Refinement

*R*[*F* ^2^ > 2σ(*F* ^2^)] = 0.030
*wR*(*F* ^2^) = 0.081
*S* = 1.05
3055 reflections
193 parameters
H-atom parameters constrained
Δρ_max_ = 0.29 e Å^−3^
Δρ_min_ = −0.65 e Å^−3^

### 

Data collection: *SMART* (Bruker, 2001 ▶); cell refinement: *SAINT* (Bruker, 2001 ▶); data reduction: *SAINT*; program(s) used to solve structure: *SHELXS97* (Sheldrick, 2008 ▶); program(s) used to refine structure: *SHELXL97* (Sheldrick, 2008 ▶); molecular graphics: *ORTEP-3* (Farrugia, 1997 ▶) and *DIAMOND* (Brandenburg, 1998 ▶); software used to prepare material for publication: *SHELXL97*.

## Supplementary Material

Crystal structure: contains datablocks I. DOI: 10.1107/S160053680802669X/ng2486sup1.cif

Structure factors: contains datablocks I. DOI: 10.1107/S160053680802669X/ng2486Isup2.hkl

Additional supplementary materials:  crystallographic information; 3D view; checkCIF report

## Figures and Tables

**Table 1:** Hydrogen-bond geometry (Å, °)

*D*—H⋯*A*	*D*—H	H⋯*A*	*D*⋯*A*	*D*—H⋯*A*
C14—H14⋯O2^i^	0.93	2.54	3.371 (3)	150

Symmetry code: (i) 
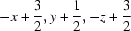
.

**Fig. 1.**
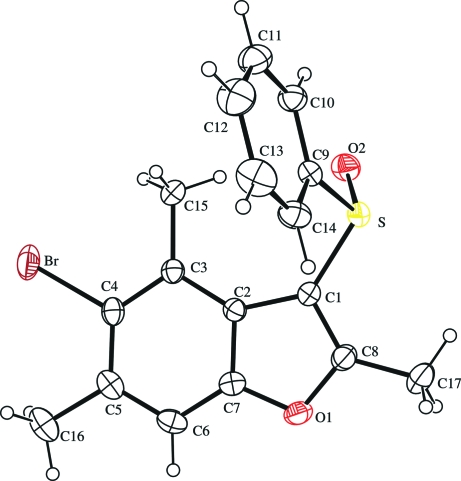
The molecular structure of the title compound, showing displacement ellipsoids drawn at the 30% probability level.

**Fig. 2.**
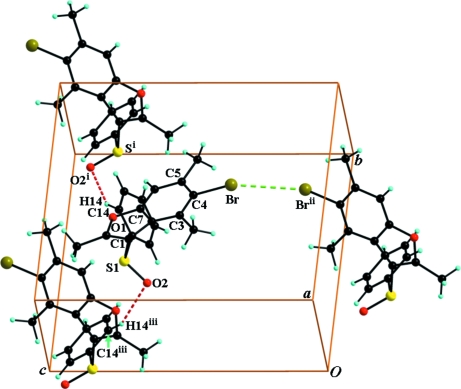
C—H···O and Br···Br interactions (dotted lines) in the title compound. [Symmetry code: (i) -x + 3/2, y + 1/2, -z + 3/2; (ii) -x + 1, y, -z + 1/2; (iii) -x + 3/2, y - 1/2, -z + 3/2.]

## supplementary crystallographic information

### Comment

This work is related to our communications on the synthesis and structures of 2-methyl-3-phenylsulfinyl-1-benzofuran analogues, *viz*. 5-bromo-2-methyl-3-phenylsulfinyl-1-benzofuran (Seo *et al.*, 2007) and 5-iodo-2,7-dimethyl-3-phenylsulfinyl-1-benzofuran (Choi *et al.*, 2008). Here we report the crystal structure of the title compound, 5-bromo-2,4,6-trimethyl-3-phenylsulfinyl-1-benzofuran (Fig. 1).

The benzofuran unit is essentially planar, with a mean deviation of 0.023 (2) Å from the least-squares plane defined by the nine constituent atoms. The phenyl ring (C9—C14) is almost perpendicular to the plane of the benzofuran ring system [75.11 (7)°]. The crystal packing (Fig. 2) is stabilized by intermolecular C—H···O interactions between a phenyl H atom of the phenylsulfinyl substituent and the oxygen of S?O unit, with a C14—H14···O2^i^ separation of 2.54 Å (Fig. 2 and Table 1; symmetry code as in Fig. 2). Further stability comes from a Br···Br^ii^ interaction at 3.7169 (6) Å (Fig. 2).

### Experimental

77% 3-Chloroperoxybenzoic acid (123 mg, 0.55 mmol) was added in small portions to a stirred solution of 5-bromo-2,4,6-trimethyl-3-phenylsulfanyl-1-benzofuran (174 mg, 0.5 mmol) in dichloromethane (20 ml) at 273 K. After being stirred at room temperature for 4 h, the mixture was washed with saturated sodium bicarbonate solution and the organic layer was separated, dried over magnesium sulfate, filtered and concentrated in vacuum. The residue was purified by column chromatography (hexane-ethyl acetate, 1:1 *v*/*v*) to afford the title compound as a colorless solid [yield 78%, m.p. 446–447 K; *R*_f_ = 0.79 (hexane-ethyl acetate, 1:1 *v*/*v*)]. Single crystals suitable for X-ray diffraction were prepared by evaporation of a solution of the title compound in benzene at room temperature. Spectroscopic analysis: ^1^H NMR (CDCl_3_, 400 MHz) δ 2.36 (s, 3H), 2.47 (s, 3H), 2.72 (s, 3H), 7.21 (s, 1H), 7.41–7.49 (m, 5H); EI—MS 364 [*M*+2], 362 [*M*^+^].

### Refinement

All H atoms were positioned geometrically and refined using a riding model, with C—H=0.93 Å for aromatic H atoms and 0.96 Å for methyl H atoms, respectively, and with *U*_iso_(H) = 1.2Ueq(C) for aromatic H atoms and 1.5Ueq(C) for methyl H atoms.

### Crystal data

**Table:**

C_17_H_15_BrO_2_S	*F*_000_ = 1472
*M**_r_* = 363.26	*D*_x_ = 1.547 Mg m^−^^3^
Monoclinic, *C*2/*c*	Melting point = 446–447 K
Hall symbol: -C_2yc	Mo *K*α radiation λ = 0.71073 Å
*a* = 22.114 (2) Å	Cell parameters from 4364 reflections
*b* = 10.4281 (8) Å	θ = 2.3–27.6º
*c* = 16.675 (1) Å	µ = 2.77 mm^−^^1^
β = 125.767 (1)º	*T* = 298 (2) K
*V* = 3120.1 (4) Å^3^	Block, colorless
*Z* = 8	0.30 × 0.20 × 0.10 mm

### Data collection

**Table:**

Bruker SMART CCD diffractometer	3055 independent reflections
Radiation source: fine-focus sealed tube	2607 reflections with *I* > 2σ(*I*)
Monochromator: graphite	*R*_int_ = 0.015
Detector resolution: 10.0 pixels mm^-1^	θ_max_ = 26.0º
*T* = 298(2) K	θ_min_ = 3.0º
φ and ω scans	*h* = −27→26
Absorption correction: multi-scan(SADABS; Sheldrick, 1999)	*k* = −9→12
*T*_min_ = 0.528, *T*_max_ = 0.762	*l* = −20→19
8646 measured reflections	

### Refinement

**Table:**

Refinement on *F*^2^	Secondary atom site location: difference Fourier map
Least-squares matrix: full	Hydrogen site location: inferred from neighbouring sites
*R*[*F*^2^ > 2σ(*F*^2^)] = 0.030	H-atom parameters constrained
*wR*(*F*^2^) = 0.081	*w* = 1/[σ^2^(*F*_o_^2^) + (0.0445*P*)^2^ + 2.4497*P*] where *P* = (*F*_o_^2^ + 2*F*_c_^2^)/3
*S* = 1.05	(Δ/σ)_max_ = 0.001
3055 reflections	Δρ_max_ = 0.29 e Å^−^^3^
193 parameters	Δρ_min_ = −0.65 e Å^−^^3^
Primary atom site location: structure-invariant direct methods	Extinction correction: none

### Special details

**Table:**

Geometry. All e.s.d.'s (except the e.s.d. in the dihedral angle between two l.s. planes) are estimated using the full covariance matrix. The cell e.s.d.'s are taken into account individually in the estimation of e.s.d.'s in distances, angles and torsion angles; correlations between e.s.d.'s in cell parameters are only used when they are defined by crystal symmetry. An approximate (isotropic) treatment of cell e.s.d.'s is used for estimating e.s.d.'s involving l.s. planes.
Refinement. Refinement of *F*^2^ against ALL reflections. The weighted *R*-factor *wR* and goodness of fit *S* are based on *F*^2^, conventional *R*-factors *R* are based on *F*, with *F* set to zero for negative *F*^2^. The threshold expression of *F*^2^ > σ(*F*^2^) is used only for calculating *R*-factors(gt) *etc*. and is not relevant to the choice of reflections for refinement. *R*-factors based on *F*^2^ are statistically about twice as large as those based on *F*, and *R*- factors based on ALL data will be even larger.

### Fractional atomic coordinates and isotropic or equivalent isotropic displacement parameters (Å^2^)

**Table:**

	*x*	*y*	*z*	*U*_iso_*/*U*_eq_	
Br	0.533506 (16)	0.68321 (3)	0.38143 (2)	0.06177 (12)	
S	0.74509 (3)	0.26302 (5)	0.71578 (4)	0.03881 (14)	
O1	0.64417 (8)	0.49816 (16)	0.78355 (11)	0.0445 (4)	
O2	0.70046 (10)	0.17705 (14)	0.62807 (13)	0.0493 (4)	
C1	0.68826 (11)	0.3831 (2)	0.71386 (15)	0.0353 (4)	
C2	0.63826 (11)	0.48016 (19)	0.64269 (15)	0.0325 (4)	
C3	0.61280 (11)	0.5170 (2)	0.54678 (15)	0.0344 (4)	
C4	0.56708 (11)	0.6251 (2)	0.51013 (16)	0.0387 (5)	
C5	0.54362 (12)	0.6941 (2)	0.55938 (18)	0.0437 (5)	
C6	0.56699 (12)	0.6518 (2)	0.65185 (18)	0.0433 (5)	
H6	0.5516	0.6927	0.6866	0.052*	
C7	0.61363 (11)	0.5474 (2)	0.69097 (15)	0.0368 (5)	
C8	0.68938 (12)	0.3992 (2)	0.79549 (16)	0.0426 (5)	
C9	0.80112 (11)	0.3609 (2)	0.69393 (15)	0.0354 (4)	
C10	0.81357 (13)	0.3179 (2)	0.62659 (18)	0.0434 (5)	
H10	0.7914	0.2426	0.5915	0.052*	
C11	0.85940 (14)	0.3879 (3)	0.6117 (2)	0.0549 (6)	
H11	0.8672	0.3608	0.5652	0.066*	
C12	0.89331 (16)	0.4973 (3)	0.6653 (2)	0.0638 (7)	
H12	0.9245	0.5438	0.6558	0.077*	
C13	0.88124 (15)	0.5388 (3)	0.7338 (2)	0.0634 (7)	
H13	0.9043	0.6132	0.7699	0.076*	
C14	0.83519 (13)	0.4705 (2)	0.74880 (17)	0.0477 (6)	
H14	0.8272	0.4979	0.7950	0.057*	
C15	0.63413 (14)	0.4441 (2)	0.48921 (17)	0.0464 (5)	
H15A	0.5921	0.4395	0.4211	0.070*	
H15B	0.6494	0.3590	0.5156	0.070*	
H15C	0.6745	0.4873	0.4941	0.070*	
C16	0.49532 (16)	0.8122 (3)	0.5165 (2)	0.0644 (8)	
H16A	0.4891	0.8484	0.5642	0.097*	
H16B	0.4475	0.7893	0.4580	0.097*	
H16C	0.5187	0.8740	0.5001	0.097*	
C17	0.73101 (17)	0.3360 (3)	0.89359 (19)	0.0639 (8)	
H17A	0.7592	0.2654	0.8942	0.096*	
H17B	0.6965	0.3049	0.9064	0.096*	
H17C	0.7643	0.3966	0.9437	0.096*	

### Atomic displacement parameters (Å^2^)

**Table:**

	*U*^11^	*U*^22^	*U*^33^	*U*^12^	*U*^13^	*U*^23^
Br	0.06404 (19)	0.0623 (2)	0.05692 (18)	0.01291 (13)	0.03418 (15)	0.02859 (12)
S	0.0454 (3)	0.0340 (3)	0.0419 (3)	0.0069 (2)	0.0283 (2)	0.0079 (2)
O1	0.0456 (8)	0.0564 (10)	0.0369 (8)	0.0034 (7)	0.0271 (7)	−0.0034 (7)
O2	0.0603 (10)	0.0352 (8)	0.0607 (11)	−0.0054 (7)	0.0400 (9)	−0.0046 (7)
C1	0.0386 (10)	0.0360 (11)	0.0338 (10)	0.0023 (9)	0.0226 (9)	0.0029 (9)
C2	0.0326 (9)	0.0303 (10)	0.0366 (10)	−0.0017 (8)	0.0214 (9)	−0.0003 (8)
C3	0.0342 (10)	0.0333 (11)	0.0367 (10)	−0.0025 (8)	0.0214 (9)	0.0011 (8)
C4	0.0359 (10)	0.0351 (11)	0.0411 (11)	−0.0027 (9)	0.0203 (9)	0.0058 (9)
C5	0.0334 (11)	0.0353 (12)	0.0542 (14)	−0.0009 (9)	0.0209 (10)	−0.0011 (10)
C6	0.0348 (11)	0.0436 (12)	0.0509 (13)	−0.0014 (9)	0.0247 (10)	−0.0110 (10)
C7	0.0342 (10)	0.0400 (11)	0.0377 (11)	−0.0045 (9)	0.0219 (9)	−0.0047 (9)
C8	0.0443 (11)	0.0491 (13)	0.0372 (11)	0.0020 (10)	0.0254 (10)	0.0040 (10)
C9	0.0354 (10)	0.0327 (10)	0.0371 (11)	0.0051 (8)	0.0206 (9)	0.0033 (9)
C10	0.0488 (13)	0.0405 (12)	0.0452 (12)	−0.0009 (10)	0.0298 (11)	−0.0044 (9)
C11	0.0593 (15)	0.0597 (16)	0.0608 (15)	−0.0007 (13)	0.0437 (13)	0.0003 (13)
C12	0.0615 (16)	0.0614 (17)	0.0824 (19)	−0.0138 (14)	0.0499 (16)	−0.0009 (15)
C13	0.0581 (15)	0.0517 (15)	0.0765 (19)	−0.0172 (13)	0.0371 (15)	−0.0170 (14)
C14	0.0487 (13)	0.0460 (13)	0.0479 (13)	−0.0011 (11)	0.0279 (11)	−0.0100 (10)
C15	0.0559 (13)	0.0511 (14)	0.0396 (12)	0.0086 (11)	0.0320 (11)	0.0078 (10)
C16	0.0557 (15)	0.0468 (15)	0.080 (2)	0.0150 (12)	0.0338 (15)	0.0080 (13)
C17	0.0720 (18)	0.082 (2)	0.0390 (13)	0.0163 (15)	0.0334 (13)	0.0139 (13)

### Geometric parameters (Å, °)

**Table:**

Br—Br^i^	3.7169 (6)	C9—C14	1.380 (3)
Br—C4	1.914 (2)	C10—C11	1.385 (3)
S—O2	1.4930 (18)	C10—H10	0.9300
S—C1	1.761 (2)	C11—C12	1.371 (4)
S—C9	1.799 (2)	C11—H11	0.9300
O1—C8	1.368 (3)	C12—C13	1.385 (4)
O1—C7	1.373 (3)	C12—H12	0.9300
C1—C8	1.357 (3)	C13—C14	1.380 (4)
C1—C2	1.456 (3)	C13—H13	0.9300
C2—C7	1.396 (3)	C14—H14	0.9300
C2—C3	1.404 (3)	C15—H15A	0.9600
C3—C4	1.394 (3)	C15—H15B	0.9600
C3—C15	1.500 (3)	C15—H15C	0.9600
C4—C5	1.400 (3)	C16—H16A	0.9600
C5—C6	1.382 (4)	C16—H16B	0.9600
C5—C16	1.509 (3)	C16—H16C	0.9600
C6—C7	1.373 (3)	C17—H17A	0.9600
C6—H6	0.9300	C17—H17B	0.9600
C8—C17	1.483 (3)	C17—H17C	0.9600
C9—C10	1.378 (3)		
			
O2—S—C1	110.68 (10)	C9—C10—H10	120.3
O2—S—C9	106.35 (10)	C11—C10—H10	120.3
C1—S—C9	99.28 (10)	C12—C11—C10	120.0 (2)
C8—O1—C7	106.46 (16)	C12—C11—H11	120.0
C8—C1—C2	106.94 (19)	C10—C11—H11	120.0
C8—C1—S	118.37 (17)	C11—C12—C13	120.2 (2)
C2—C1—S	134.65 (15)	C11—C12—H12	119.9
C7—C2—C3	119.17 (19)	C13—C12—H12	119.9
C7—C2—C1	104.22 (17)	C14—C13—C12	120.4 (2)
C3—C2—C1	136.61 (19)	C14—C13—H13	119.8
C4—C3—C2	115.22 (19)	C12—C13—H13	119.8
C4—C3—C15	123.21 (19)	C13—C14—C9	118.8 (2)
C2—C3—C15	121.57 (19)	C13—C14—H14	120.6
C3—C4—C5	125.5 (2)	C9—C14—H14	120.6
C3—C4—Br	117.02 (16)	C3—C15—H15A	109.5
C5—C4—Br	117.45 (16)	C3—C15—H15B	109.5
C6—C5—C4	117.7 (2)	H15A—C15—H15B	109.5
C6—C5—C16	119.3 (2)	C3—C15—H15C	109.5
C4—C5—C16	123.0 (2)	H15A—C15—H15C	109.5
C7—C6—C5	118.1 (2)	H15B—C15—H15C	109.5
C7—C6—H6	121.0	C5—C16—H16A	109.5
C5—C6—H6	121.0	C5—C16—H16B	109.5
O1—C7—C6	124.74 (19)	H16A—C16—H16B	109.5
O1—C7—C2	111.03 (18)	C5—C16—H16C	109.5
C6—C7—C2	124.2 (2)	H16A—C16—H16C	109.5
C1—C8—O1	111.34 (19)	H16B—C16—H16C	109.5
C1—C8—C17	133.4 (2)	C8—C17—H17A	109.5
O1—C8—C17	115.2 (2)	C8—C17—H17B	109.5
C10—C9—C14	121.2 (2)	H17A—C17—H17B	109.5
C10—C9—S	118.02 (17)	C8—C17—H17C	109.5
C14—C9—S	120.60 (17)	H17A—C17—H17C	109.5
C9—C10—C11	119.4 (2)	H17B—C17—H17C	109.5
			
O2—S—C1—C8	127.65 (18)	C5—C6—C7—O1	176.8 (2)
C9—S—C1—C8	−120.85 (19)	C5—C6—C7—C2	−0.9 (3)
O2—S—C1—C2	−55.0 (2)	C3—C2—C7—O1	179.99 (17)
C9—S—C1—C2	56.5 (2)	C1—C2—C7—O1	−0.4 (2)
C8—C1—C2—C7	0.1 (2)	C3—C2—C7—C6	−2.0 (3)
S—C1—C2—C7	−177.40 (18)	C1—C2—C7—C6	177.5 (2)
C8—C1—C2—C3	179.6 (2)	C2—C1—C8—O1	0.2 (3)
S—C1—C2—C3	2.0 (4)	S—C1—C8—O1	178.23 (15)
C7—C2—C3—C4	3.4 (3)	C2—C1—C8—C17	−177.0 (3)
C1—C2—C3—C4	−176.0 (2)	S—C1—C8—C17	1.1 (4)
C7—C2—C3—C15	−176.74 (19)	C7—O1—C8—C1	−0.5 (2)
C1—C2—C3—C15	3.9 (4)	C7—O1—C8—C17	177.2 (2)
C2—C3—C4—C5	−2.3 (3)	O2—S—C9—C10	−20.6 (2)
C15—C3—C4—C5	177.9 (2)	C1—S—C9—C10	−135.45 (18)
C2—C3—C4—Br	177.91 (14)	O2—S—C9—C14	164.37 (18)
C15—C3—C4—Br	−1.9 (3)	C1—S—C9—C14	49.5 (2)
C3—C4—C5—C6	−0.5 (3)	C14—C9—C10—C11	−1.9 (3)
Br—C4—C5—C6	179.28 (16)	S—C9—C10—C11	−176.89 (19)
C3—C4—C5—C16	178.7 (2)	C9—C10—C11—C12	1.5 (4)
Br—C4—C5—C16	−1.5 (3)	C10—C11—C12—C13	−0.7 (4)
C4—C5—C6—C7	2.1 (3)	C11—C12—C13—C14	0.1 (5)
C16—C5—C6—C7	−177.1 (2)	C12—C13—C14—C9	−0.4 (4)
C8—O1—C7—C6	−177.4 (2)	C10—C9—C14—C13	1.3 (4)
C8—O1—C7—C2	0.6 (2)	S—C9—C14—C13	176.2 (2)

Symmetry codes: (i) −*x*+1, *y*, −*z*+1/2.

### Hydrogen-bond geometry (Å, °)

**Table:**

*D*—H···*A*	*D*—H	H···*A*	*D*···*A*	*D*—H···*A*
C14—H14···O2^ii^	0.93	2.54	3.371 (3)	150
C15—H15A···Br	0.96	2.75	3.118 (2)	103

Symmetry codes: (ii) −*x*+3/2, *y*+1/2, −*z*+3/2.
